# Fidaxomicin vs Vancomycin for the Treatment of a First Episode of Clostridium Difficile Infection: A Meta-analysis and Systematic Review

**DOI:** 10.7759/cureus.2778

**Published:** 2018-06-11

**Authors:** Laith A Al Momani, Omar Abughanimeh, Boonphiphop Boonpheng, Joseph Gabriel Gabriel, Mark Young

**Affiliations:** 1 Department of Internal Medicine, East Tennessee State University, Johnson City, USA; 2 Department of Internal Medicine, University of Missouri Kansas City School of Medicine, Kansas City, USA; 3 Department of Gastroenterology, East Tennessee State University, Johnson City, USA

**Keywords:** clostridium difficile, treatment of clostridium difficile, vancomycin, fidaxomicin, c. diff, c. difficile

## Abstract

Clostridium difficile infection (CDI) continues to possess a significant disease burden in the United States (US) as well as all over the world. Given the increase in severity and recurrence rate, the decrease in cure rate, and the fact that the virulent ribotype 027 strain remains one of the most commonly identified strains in the US, the Infectious Diseases Society of America (IDSA) published a clinical practice guideline in February 2018 moving away from metronidazole as the first-line treatment for initial CDI and recommending either oral vancomycin or fidaxomicin. The aim of this study is to evaluate the clinical data available comparing the efficacy of primary treatment of CDI between those two antibiotics. We performed a PubMed, PubMed Central, and ScienceDirect database search without restriction to regions, publication types, or languages. A comprehensive literature search was performed from January 1, 1980 up to March 20, 2018. We used the following keywords in different combinations: Clostridium difficile, Clostridium difficile infection, CDI, C. diff, C. difficile, fidaxomicin, vancomycin, pseudomembranous colitis, and antibiotic-associated colitis. The search was limited to human studies. Data were independently extracted by two reviewers with disagreements resolved by a third author. We pooled an odds ratio (OR) on two primary outcomes: Clinical cure rate and rate of recurrence during the follow-up period. The computer search was also supplemented with manual searches by the authors of the retrieved review articles and primary studies. The search phrase “((Clostridium difficile) AND vancomycin) AND fidaxomicin” had the highest yield results. We identified four observational studies with a total of 2,303 patients with CDI that met our inclusion criteria. Compared with vancomycin, fidaxomicin use was associated with a significantly lower recurrence of CDI with a pooled OR of 0.47 (95% confidence interval (CI), 0.37 - 0.60, I2 = 0). On the other hand, there was no significant association of fidaxomicin use with CDI cure rate compared to vancomycin with a pooled OR of 1.22 (95% CI, 0.93 - 1.60, I2 = 0). In light of the recently updated clinical practice guidelines by the IDSA, our review suggests that fidaxomicin has a more sustained clinical response with a statistically significant lower recurrence rate. Although fidaxomicin appears to be the better drug with statistical significance, its cost-effectiveness continues to be an ongoing controversy. More randomized clinical trials are needed to shed light on this matter to assess if there is any clinical significance in fidaxomicin superiority.

## Introduction and background

Clostridium difficile infection (CDI) continues to possess a significant disease burden in both the United States (US) and globally. There have been a reported 453,000 infections in 2011 in the US alone, with 83,000 of those experiencing at least one recurrence and 29,000 expiring within 30 days of the initial diagnosis [1]. The incidence and severity of CDI continue to trend upwards, with a reported increase in community-acquired infections of a disease that was once considered nosocomial and antibiotic-related [2-4]. A similar trend is reported in children [5-6].

Due to its severity and high rates of recurrence, lower cure rates, and ribotype 027 virulence in the US, the Infectious Diseases Society of America (IDSA) published clinical practice guidelines in February 2018 moving away from metronidazole as the first-line treatment for initial CDI and recommending, with a strong level of evidence, either oral vancomycin or oral fidaxomicin [7].

The purpose of this systematic review and meta-analysis is to analyze the available data on the comparison of oral vancomycin and oral fidaxomicin as the first-line medication treatment of CDI. The reader must bear in mind that multiple studies have attempted to prove that although fidaxomicin is more costly than the alternatives, it may prove to be the more cost-effective option.

## Review

Methods

Search Strategy and Selection Criteria

We performed a PubMed, PubMed Central, and ScienceDirect database search without restriction to regions, publication types, or languages. A comprehensive literature search was performed from inception to March 20, 2018, during which time the IDSA guidelines update on changing the initial drug of choice to oral vancomycin or fidaxomicin has been set forth. The following keywords were used in different combinations: Clostridium difficile, Clostridium difficile infection, CDI, C. diff, C. difficile, fidaxomicin, vancomycin, pseudomembranous colitis, and antibiotic-associated colitis. The search was limited to human studies. The computer search was supplemented with manual searches by the authors of the retrieved review articles and primary studies. The search phrase ((Clostridium difficile) AND vancomycin) AND fidaxomicin had the highest yield results.

Data Extraction and Quality Assessment

Studies were included if they met the following: 1) used a well-defined case-control or cohort design and 2) presented an odds ratio (OR) for our main outcome with a 95% confidence interval (CI) or reported sufficient data to calculate these parameters. Exclusion criteria were: 1) case reports, case series, and review articles and 2) insufficient information concerning evaluation rates. Two authors screened all abstracts independently obtained from the initial literature search and removed those not fulfilling the inclusion criteria. The data were abstracted from all included studies into a standardized table. A third investigator reviewed the data for accuracy prior to the data query. The inclusion/exclusion decisions were made after consultation with the other authors.

Outcome Definition

In comparing the efficacy of treatment of initial CDI with vancomycin versus fidaxomicin, two endpoints were used: 1) achieving clinical cure with a resolution of symptoms without the need for further treatment and 2) recurrence of infection during the follow-up period, which was at least three weeks and up to four weeks following a 10-day course of antibiotics. This included relapse and reinfection. An overall OR was used for both of these endpoints.

Statistical Analysis

All statistical analyses were performed using the Comprehensive Meta-Analysis (CMA), Version 3 software (BioStat, Inc., Eaglewood, NJ). The pooled risk ratios of Clostridium difficile cure and recurrence in patients treated with fidaxomicin in comparison to those treated with vancomycin were calculated using the generic inverse method of DerSimonian and Laird [8]. A random effect model was used, given the high likelihood of between-study variance due to the difference in underlying population and methodology. Cochran's Q-test, which was supplemented by I^2^ statistic, was used to evaluate the statistical heterogeneity. This I^2^ statistic quantifies the proportion of the total variation across studies, that is, due to true heterogeneity rather than chance. A value of I^2^ of 0% to 25% denotes trivial heterogeneity, greater than 25% but ≤ 50% denotes low heterogeneity, greater than 50% but ≤ 75% denotes moderate heterogeneity, and greater than 75% represents high heterogeneity [9].

Results

Search Results

**Figure 1:**
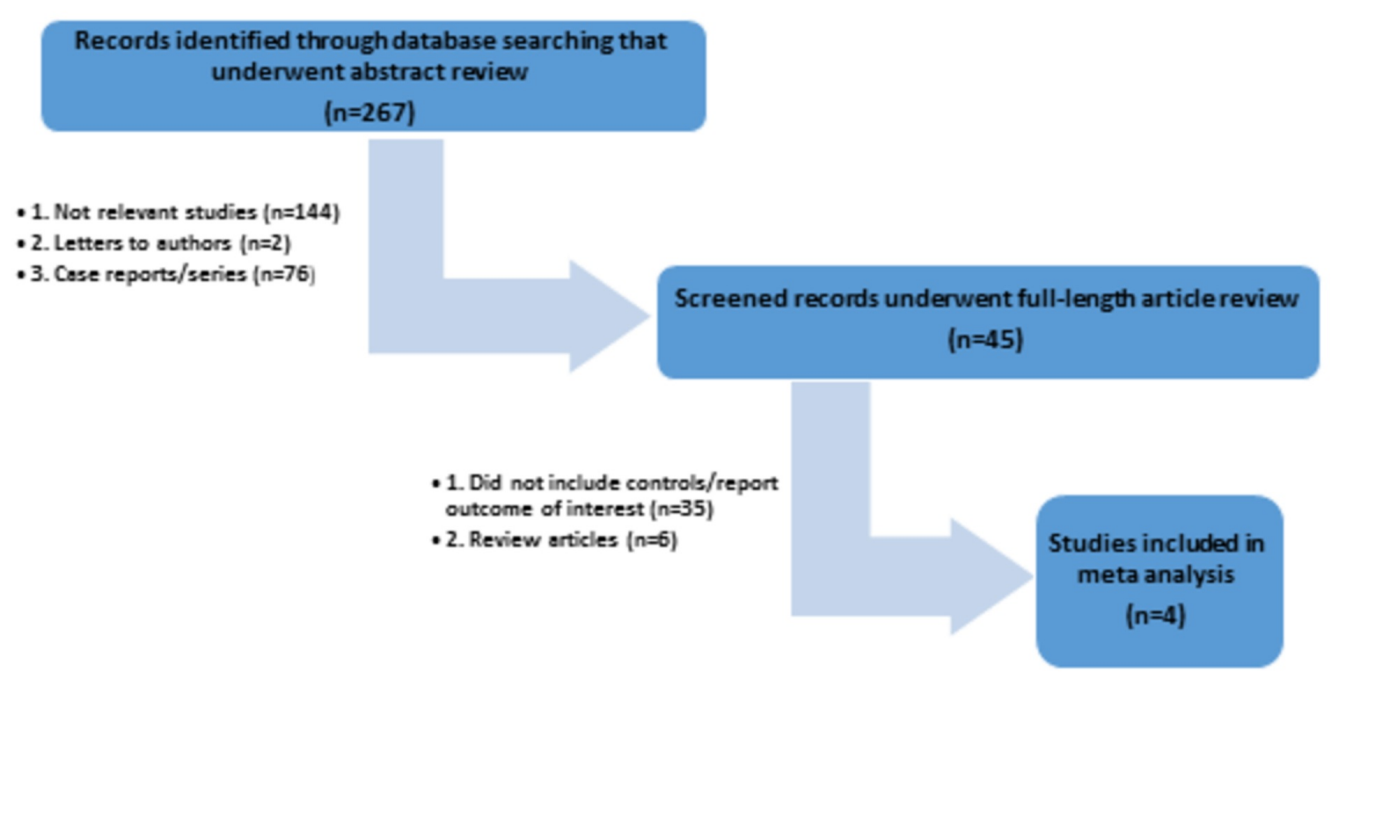
A flow diagram demonstrates the search methodology and selection process for this meta-analysis n: number

The initial search yielded 267 citations, all of which underwent title and abstract review. The majority of them were excluded at this step, including those that were case reports, letters to editor, review articles, or interventional studies.  A total of 45 studies underwent full-length article review, and 41 of them were excluded as they did not include controls, did not report the outcome of interest, or were review articles. Therefore, a total of four studies [10-13] met our inclusion criteria and were included in the meta-analysis. Figure 1 outlines our search methodology and selection process. Baseline characteristics of the included studies are summarized in Table 1.

**Table 1:** Total Studies Utilized in This Meta-analysis BMI: body mass index; CDI: Clostridium difficile infection; NAP1: North American pulsed-field gel electrophoresis type 1; OR: odds ratio; UK: United Kingdom; US: United States; WBC: white blood cells

	Louie et al. [12]	Cornely et al. [13]	Loui et al. [10]	Houseman et al. [11]
Country	US and Canada	US, Canada, France, Spain, Belgium, Germany, UK, Italy, Sweden	US, Canada, France, Spain, Belgium,Germany, UK, Italy, Sweden	US
Study design	Clinical trial	Clinical trial	Clinical trial	Clinical trial
Year	2011	2012	2013	2016
Number of participants enrolled	629	535	1,105	34
Number of participants enrolled/Fidaxomicin	302	271	539	18
Number of participants enrolled/Vancomycin	327	264	566	16
Overall participants analyzed	596	509	794	24
Overall participants analyzed/Fidaxomicin	287	253	391	12
Overall participants analayzed/Vancomycin	309	256	403	12
Mean age of participants in years	61.9	63.4	Fidaxomicin: 63.3 Vancomycin: 62.3	Fidaxomicin: 69 Vancomycin: 66
Median follow up duration	21 days	21 days	21 days	28 days
OR: Cure rate, Fidaxomicin vs Vancomycin	1.24 (0.77 - 2.00)	1.09 (0.65 - 1.83)	1.24 (0.8 - 1.92)	2.5 (0.46 - 13.52)
OR: Recurrence rate, Fidaxomicin vs Vancomycin	0.54 (0.35 - 0.84)	0.39 (0.24 - 0.64)	0.46 (0.32 - 0.67)	0.85 (0.1 - 7.04)
Confounder adjustment	Age, Sex, Inpatient status, NAP1 strain	Age, Sex, Inpatient status, NAP1 strain	Age, Sex, Inpatient status, NAP1 strain, BMI, WBC, serum albumin, serum creatinine, concomitant antibiotic therapy	Age, Sex, Community acquired CDI, NAPI strain
Quality assessment (Newcastle-Ottawa scale)	Good	Good	Good	Good

Meta-analysis results

Recurrence Rate

**Figure 2:**
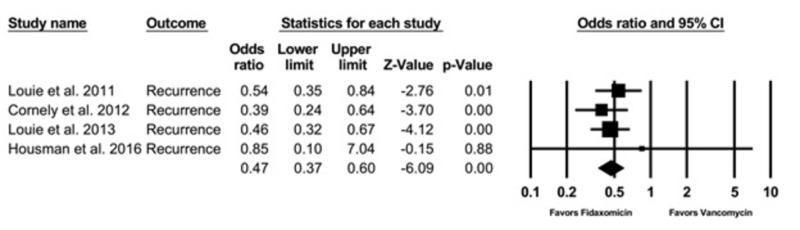
Recurrence rate CI: confidence interval

Four observational studies with a total of 2,303 patient with CDI were enrolled. Compared with vancomycin, fidaxomicin use was associated with a significantly lower recurrence with a pooled OR of 0.47 (95% CI, 0.37 - 0.60, I2 = 0) (Figure 2).

Cure Rate

**Figure 3:**
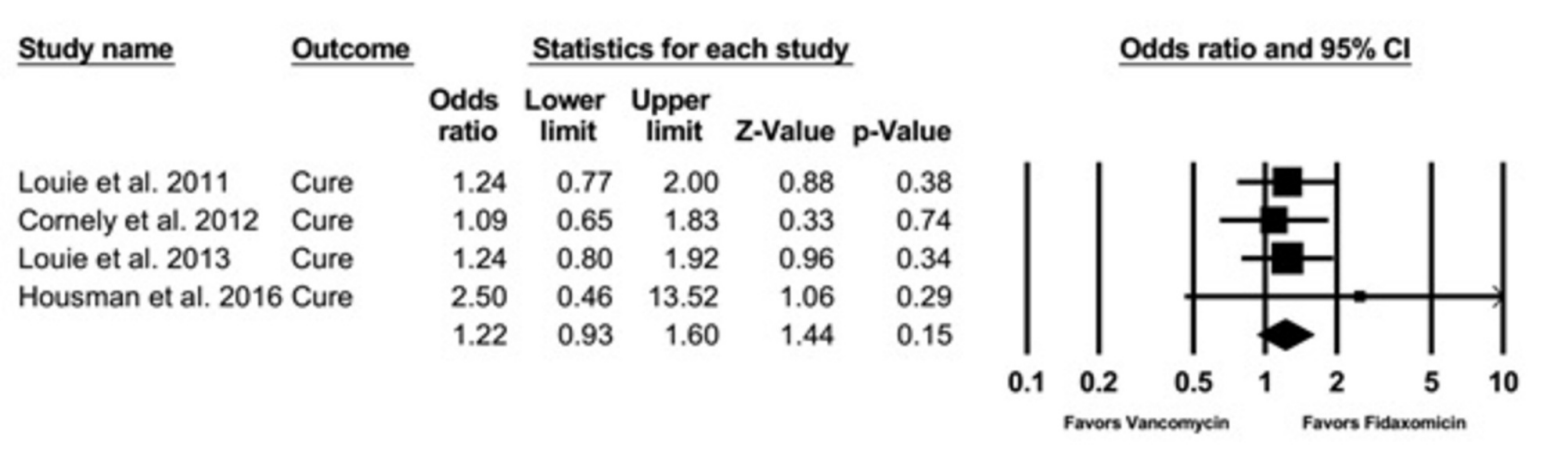
Cure rate CI: confidence interval

There was no significant association of fidaxomicin use with CDI cure rate compared to vancomycin with a pooled OR of 1.22 (95% CI, 0.93 - 1.60, I2 = 0) (Figure 3).

Evaluation for Publication Bias

**Figure 4:**
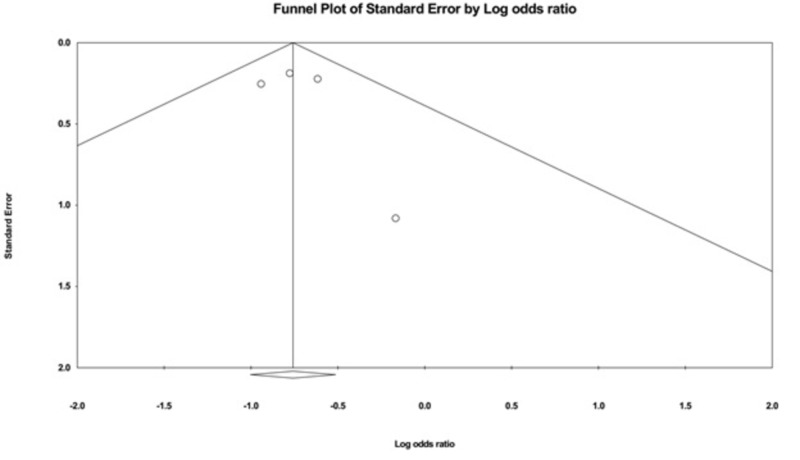
A funnel plot: recurrence rate

**Figure 5:**
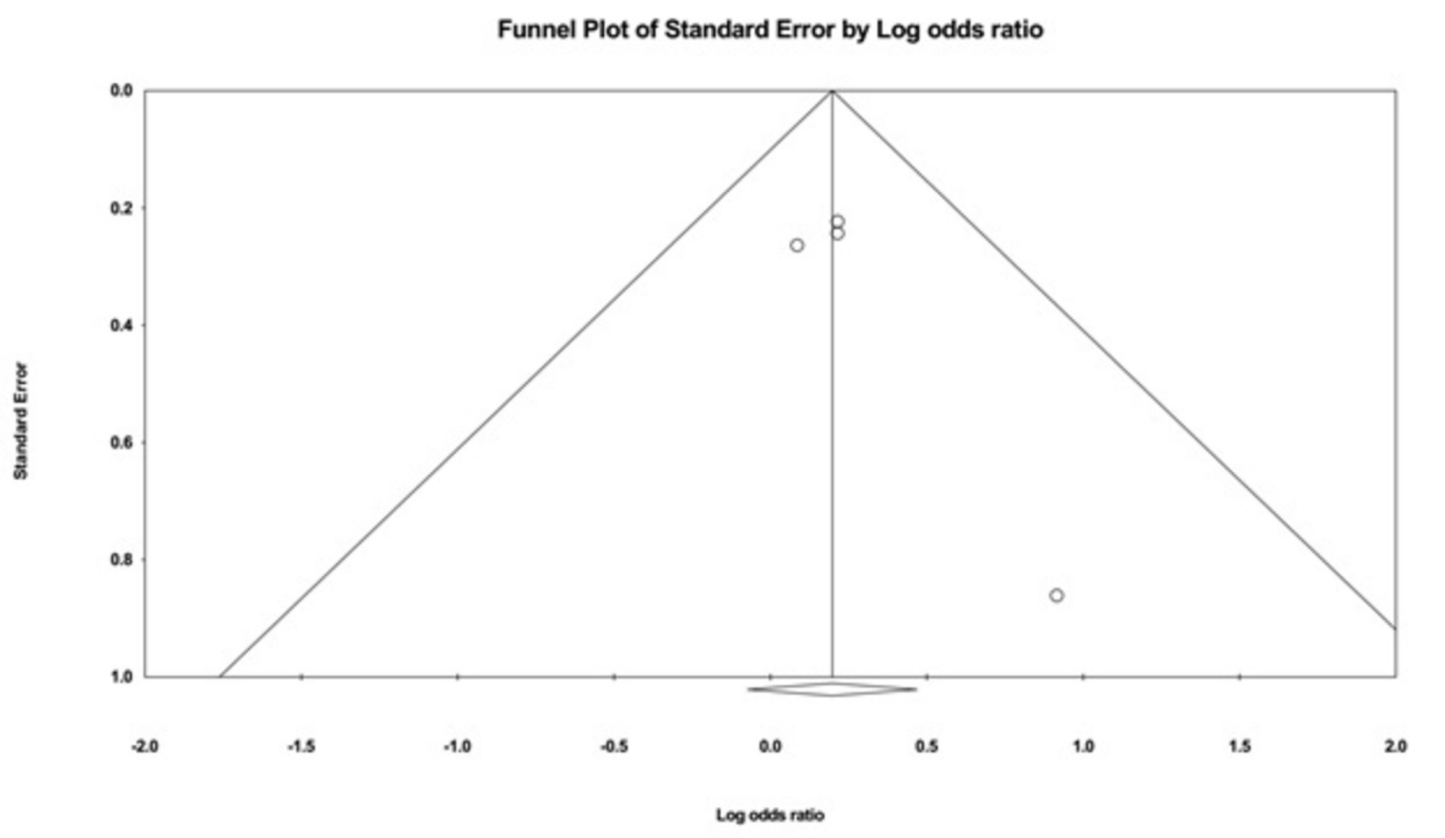
A funnel plot: cure rate

The funnel plots evaluating recurrence and cure rate are shown in Figures 4-5. They are symmetric and do not suggest the presence of publication bias in favor of a positive study for all of the outcomes. In addition, Egger’s regression asymmetry test showed no evidence of publication bias (P > 0.05 for both outcomes).

Sensitivity Analysis

Sensitivity analysis was performed by excluding one study at a time to investigate the effect of each study on the pooled odds ratio for each outcome assessed. The pooled effect estimate from this sensitivity analysis remained essentially unchanged.

Discussion

The studies we used in our meta-analysis included patients with their first episode of CDI. All of these patients had clinical symptoms suggestive of CDI, including a minimum of three episodes of unformed stool in a 24-hour period with no known or documented the prior diagnosis of CDI. The diagnosis was made by a positive C. difficile polymerase chain reaction test or toxin A, B, C assay, depending on the specific study criteria. These patients were then randomized for treatment with vancomycin, 124 mg orally four times daily, or fidaxomicin, 200 mg orally twice a day, for a total of 10 days. These patients were followed up for a minimum of three weeks and up to four weeks after completion of their antibiotic course.

In our review and meta-analysis of two endpoints (cure rate or the rate of symptoms’ resolution and the recurrence of symptoms within the follow-up period), we can show that the use of fidaxomicin was associated with a statistically significant lower recurrence. However, there was no significant difference with the cure rate when compared with that of vancomycin.

Fidaxomicin is the first macrolide antibacterial agent approved for the treatment of CDI [14]. It inhibits transcription by binding to the deoxyribonucleic acid (DNA)-template ribonucleic acid (RNA) polymerase sigma subunit and hence, prevents the initial separation of the bacterial DNA strands. Essentially, it inhibits the initiation of RNA synthesis very early on in that pathway [15-16]. This unique mechanism of action might explain fidaxomicin’s very narrow spectrum of antimicrobial coverage at low concentrations [17-19].

Multiple studies have shown fidaxomicin to have a substantially higher in vitro activity against C. difficile compared to vancomycin [20-23] with a more prolonged post-antibiotic effect [24]. Furthermore, fidaxomicin is a bactericidal drug, whereas vancomycin is bacteriostatic [25]. Fidaxomicin’s lower rates of relapse following treatment might be attributed to the fact that it causes fewer changes to the bowel microbiota of C. difficile-infected patients compared to vancomycin both during [26-27] and after treatment [28]. Fidaxomicin also has a narrower antimicrobial coverage [17], and unlike vancomycin, it inhibits sporulation. As Louie et al. [29] showed, patients treated with fidaxomicin had a 2.3 log10 lower fecal spore counts at 21 - 28 days post-therapy compared to a patient treated with vancomycin. Similar results were reported by Housman et al. years later [11]. This might at least suggest that fidaxomicin might be a better first-line option for patients at a higher risk of recurrence, like older patients or patients with cancer [10]. It is worth noting here that a study by Nerandzic et al. in 2012 showed that fidaxomicin reduced acquisition and overgrowth of vancomycin-resistant enterococci and Candida species in CDI patients compared to vancomycin [30].

Similar to vancomycin, fidaxomicin demonstrates minimal systemic absorption, which explains why the most common side effects are gastrointestinal in nature, like nausea and abdominal pain, both of which are likely to be part of CDI symptomatology [31-33]. This characteristic makes it a very well-tolerated drug in both adults and children [32-37].

Whenever fidaxomicin is compared with vancomycin, the cost is always an examined variable. A 10-day course has at least three times more acquisition cost than that of vancomycin [38]. However, multiple studies in high-income countries have shown the cost-effectiveness of fidaxomicin [39-41]. A recent systemic review by Burton et al. in 2017 also showed that fidaxomicin was, in fact, more cost-effective than vancomycin [42]. Prior to that, Stranges et al. performed a cost-utility analysis in the US in 2013 which showed an incremental cost-effectiveness ratio of $67,576/quality adjusted life-year (QALY) confirming the results of Sclar et al. in 2012, who also showed potential cost-effectiveness in the US health system [43-44]. Furthermore, a study with regards to hospital cost savings in 2015 showed that their hospital saved $3,047 USD (United States dollars) per patient treated with fidaxomicin compared to a patient treated with vancomycin [45]. Similar promising results were seen in patients with cancer and patients with renal impairment [41, 46]. This postulated cost-effectiveness might be explained by the decreased recurrence rate and hence, hospital readmissions that fidaxomicin provides, both of which, in addition to the length of stay, have not been well compared.

On the other hand, other studies have shown conflicting results. Reveles et al. found similar total costs comparing the two drugs [47]. Another systemic review by Le et al. in 2018 concluded that the cost-effectiveness of fidaxomicin compared to vancomycin was not definitive [48]. Costs will vary between healthcare systems as well; an increase in cost with the use of fidaxomicin has been suggested in the Canadian health care system [49].

All in all, this calls for research and strong randomized control trials to assess the cost-effectiveness of fidaxomicin compared to vancomycin in the US.

## Conclusions

In light of the recently updated clinical practice guidelines by the IDSA for the treatment of C. difficile infection, we set out to compare both of the recommended first-line drugs, vancomycin and fidaxomicin. Our meta-analysis found a similar cure rate; however, fidaxomicin was found to have a more sustained clinical response with a statistically significant lower recurrence rate. Although fidaxomicin appears to be the better drug with statistical significance, its cost-effectiveness continues to be an ongoing controversy. More randomized clinical trials are needed to shed light on this matter to assess if there is any clinical significance in fidaxomicin superiority, particularly as CDI incidence, severity, and recurrence rates continue to be on an upward trajectory.
